# Supplementary material for: β-HPV 8E6 Attenuates ATM and ATR Signaling in Response to UV Damage
Source: Pathogens. 2019 Nov 26;8(4):267. doi: 10.3390/pathogens8040267 (PMC6963835; doi:10.3390/pathogens8040267)
Supplement: Supplementary file 1 [file pathogens-08-00267-s001.zip › pathogens-616219-supplementrary final/Supplemental Figure Legends accepted.pdf]

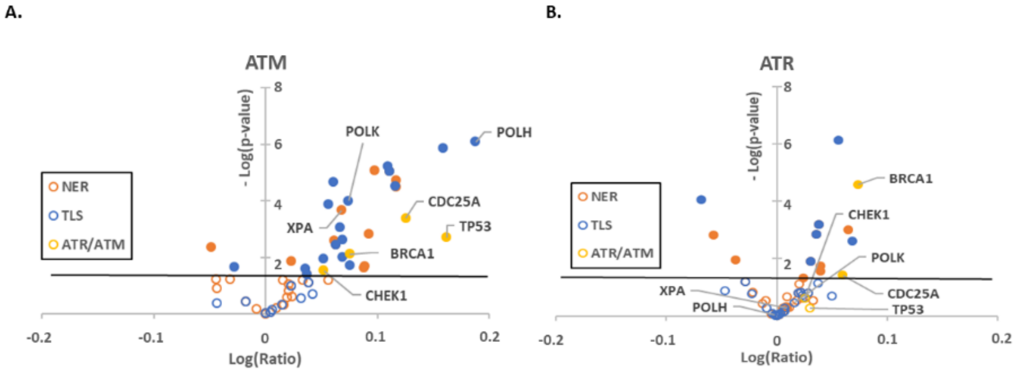

Supplemental Figure 1. High expression of ATR/ATM mRNA correlates with an increase in UV damage repair pathways gene expression. Volcano plots comparing RNAseq data of NER (orange), TLS (blue) and ATR/ATM target (yellow) genes between cell lines (a) with high ATM expression (z-score >2) and without elevated ATM expression (z-score <2) or (b) between cells with (z-score >2) and without (z-score <2) high ATR expression. Outlined circles represent non-significant expression changes. Filled in circles represent significant expression changes. Black line represents significance cutoff ($p<0.05$). The x-axis depicts the log of the ratio of each gene's expression levels in cell lines with high expression of ATM/ATR versus all other cell lines in the cancer cell line encyclopedia. The y-axis shows the negative log of the p-value. Genes with reduced expression appear to the left of the y-axis, while genes with increased expression are on the right.

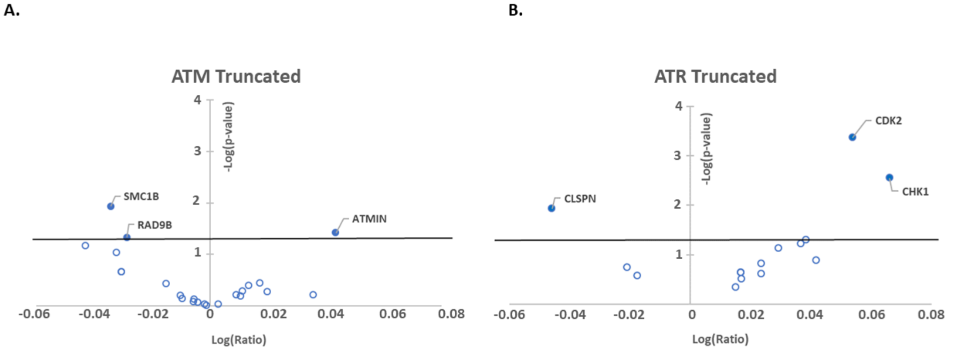

Supplemental Figure 2. Truncation of ATR/ATM weakly correlates with UV damage repair pathways gene expression. Volcano plots comparing RNAseq data of NER (orange), TLS (blue) and ATR/ATM target (yellow) genes between cell lines (a) with or without truncating ATM mutations and (b) between cell lines with and without truncating ATR mutations. Outlined circles represent non-significant expression changes. Filled in circles represent significant expression changes. Black line represents significance cutoff ($p<0.05$). The x-axis depicts the log of the ratio

of each gene's expression levels in cell lines with truncating ATM/ATR mutations versus all other cell lines in the cancer cell line encyclopedia. The y-axis shows the negative log of the p-value. Genes with reduced expression appear to the left of the y-axis, while genes with increased expression are on the right.

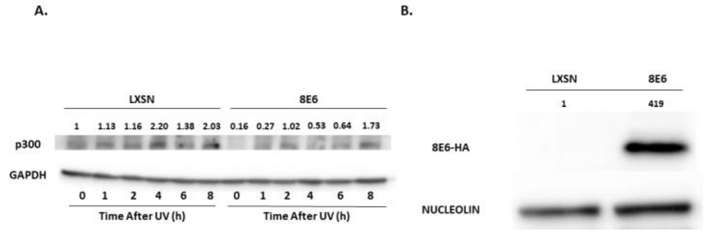

Supplemental Figure 3. Confirmation of β-HPV 8E6 expression. (a) Representative immunoblot of vector control (LXSN) and β-HPV 8E6 expressing hTERT HFKs. β-HPV 8E6 was detected indirectly via probing for its HA tag. Nucleolin was used as a loading control. (b) Representative immunoblot of untreated vector control (LXSN) and β-HPV 8E6 expressing primary HFKs. Reduced p300 served as a functional indication of β-HPV 8E6. GAPDH was used as a loading control. (a-b) The numbers above bands represent quantification by densitometry. This is shown relative to untreated cells within the vector control cell line and normalized to the loading control.

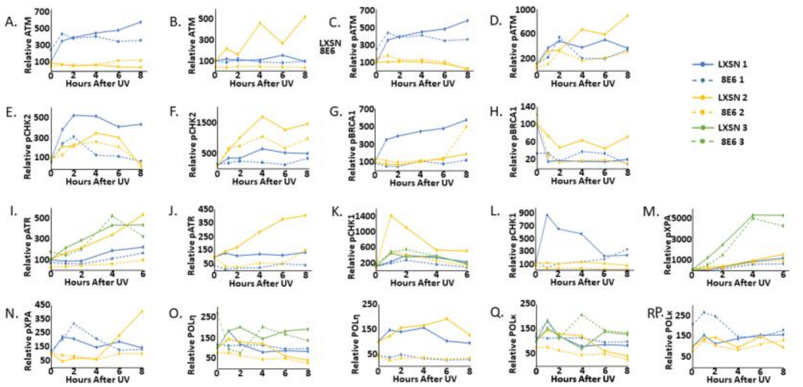

Supplemental Figure 4. Quantification of hTERT HFK and primary HFK UV time course immunoblots. Densitometry of (a) ATM in hTERT HFKs, (b) ATM in HFK, (c) P-ATM in hTERT HFKs, (d) P-ATM in HFKs, (e) P-CHK2 in hTERT HFKs, (f) P-ATM in HFKs, (g) P-BRCA1 in hTERT HFKs, (h) P-BRCA1 in primary HFKs, (i) P-ATR in hTERT HFKs, (j) P-ATR in HFKs, (k) P-CHK1 in hTERT HFKs, (l) P-CHK1 in HFKs, (m) P-XPA in hTERT HFKs, (n) P-XPA in HFKs, (o) POLη in hTERT HFKs, (p) POLη in HFKs, (q) POLκ in hTERT HFKs and (r) POLκ in HFKs. (a-r) Immunoblot quantification was done using ImageJ. LXSN (solid lines, LXSN 1-3) and 8E6 (dashed lines, 8E6 1-3) replicates are graphed individually.

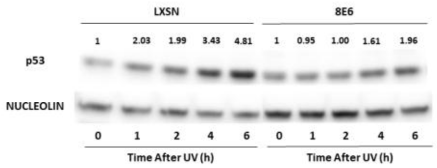

Supplemental Figure 5. β-HPV 8E6 reduces p53 accumulation after UV exposure. Representative immunoblots of hTERT HFKs with vector control (LXSN) and β-HPV 8E6 harvested 0-6 hours post 5mJ/cm² UVR. Nucleolin was used as a loading control. The numbers above bands represent quantification by densitometry. This is shown relative to untreated cells within the same cell line and normalized to the loading control.

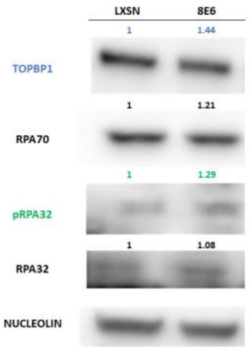

Supplemental Figure 6. β-HPV 8E6 increases phosphorylation of RPA32. Representative immunoblot of untreated vector control (LXSN) and β-HPV 8E6 expressing hTERT HFKs. Nucleolin was used as a loading control. The numbers above bands represent quantification by densitometry. This is shown relative to untreated cells within the same cell line and normalized to the loading control.

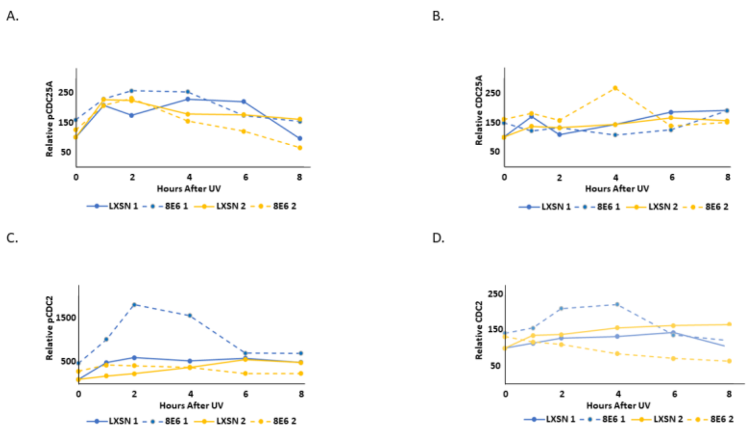

Supplemental Figure 7. Quantification of hTERT HFK UV time course immunoblots for CHK1 downstream targets. (a) P-CDC25A densitometry. (b) CDC25A densitometry. (c) P-CDC2 densitometry. (d) CDC2 densitometry. (a-d) Immunoblot quantification was done using ImageJ. LXSN (solid lines, LXSN 1-3) and 8E6 (dashed lines, 8E6 1-3) replicates are graphed individually.

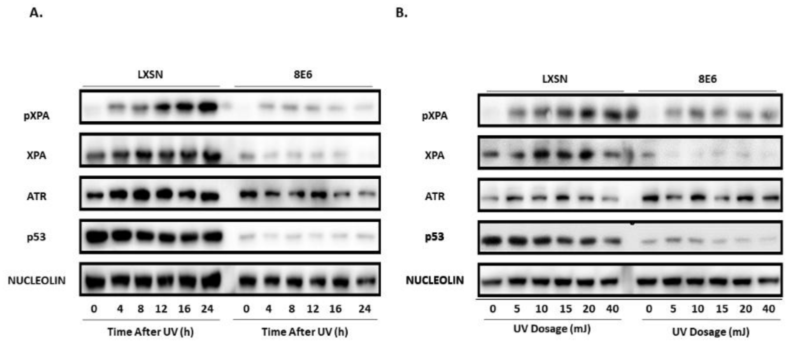

Supplemental Figure 8. β-HPV 8E6 attenuates XPA phosphorylation in U2OS cells. (a) Representative immunoblot of vector control (LXSN) and β-HPV 8E6 expressing U2OS cells harvested 4-24 hours post 15mJ/cm² UV. Nucleolin was used as a loading control. (b) Representative immunoblots of vector control (LXSN) and β-HPV 8E6 expressing U2OS cells harvested one-hour post 5-40 mJ/cm² harvested four hours post radiation UV. Nucleolin was used as a loading control.

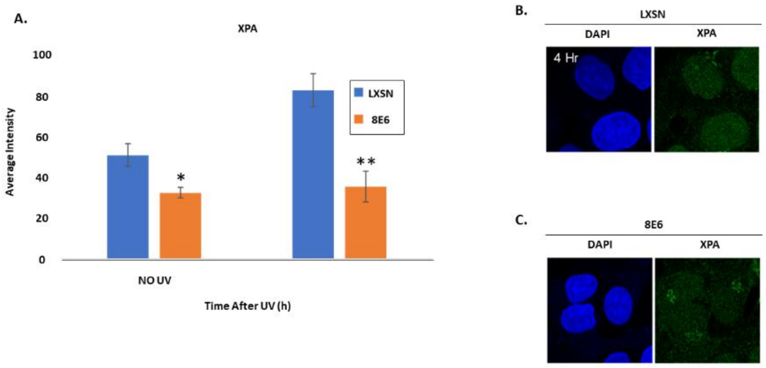

Supplemental Figure 9. β-HPV 8E6 attenuates XPA localization in U2OS cells. (a) Representative immunofluorescent images of untreated vector control (LXSN) and β-HPV 8E6 expressing U2OS 4 hours post 15 mJ/cm² UV radiation. Immunofluorescent antibodies for XPA (green) foci at 1:1000 overnight was observed with DAPI staining of the nucleus (blue).

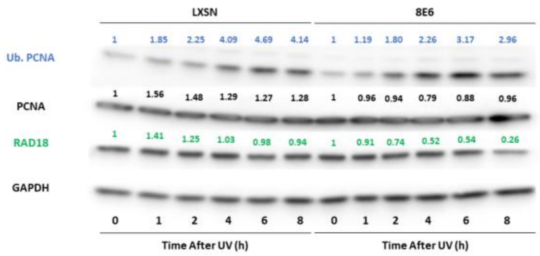

Supplemental Figure 10. β-HPV 8E6 does not attenuate other TLS proteins. Representative immunoblot of vector control (LXSN) and β-HPV 8E6 hTERT HFKs harvested 0-8 hours post 5mJ/cm² UV. GAPDH was used as a loading control. The numbers above bands represent quantification by densitometry. This is shown relative to untreated cells within the same cell line and normalized to the loading control.

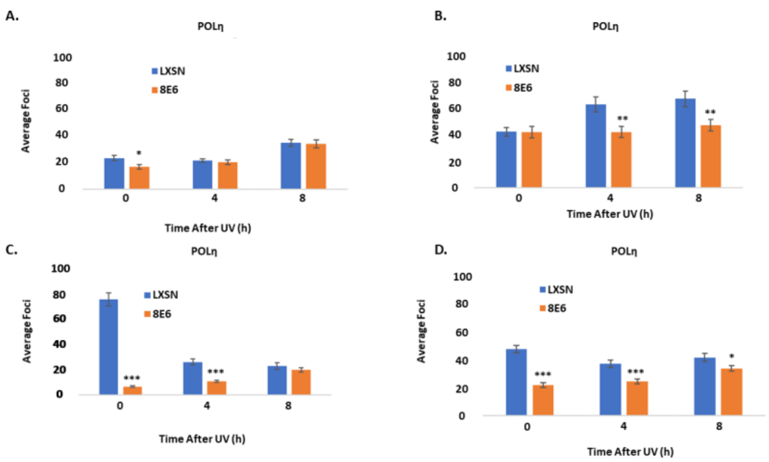

Supplemental Figure 11. β-HPV 8E6 diminished POLη foci formation and intensity. (a) Average POLη foci for the first repeat of our immunofluorescent assays. Standard error calculations were performed. (b) Average POLη foci for the second repeat of our immunofluorescent assays. Standard error calculations were performed. (c) Average POLη foci for the third repeat of our immunofluorescent assays. Standard error calculations were performed.

Supplemental Table 1. Multiple comparison (False discovery rate). Gene names and q values are listed for genes that are significant after correction for false discovery.

Gene	Q-Value	Gene	Q-Value	Gene	Q-Value
**CCNH**	5.81e-3	**LIG1**	4.83e-2	**PRIMPOL**	4.42e-3
**CDK7**	3.09e-2	**RAD18**	4.2e-3	**REV1**	3.18e-2
**ERCC1**	5.3e-3	**PCLAF**	3.05e-2	**REV3L**	1.05e-4
**ERCC4**	2.46e-4	**POLH**	1.0e-2	**RFC1**	1.10e-5
**GTF2H1**	1.55e-6	**POLI**	1.42e-4	**XPA**	1.13e-3
**GTF2H5**	6.56e-6	**POLK**	3.34e-4	**XPC**	1.75e-4
